# Centenarians as super-controls to assess the biological relevance of genetic risk factors for common age-related diseases: A proof of principle on type 2 diabetes

**DOI:** 10.18632/aging.100562

**Published:** 2013-05-31

**Authors:** Paolo Garagnani, Cristina Giuliani, Chiara Pirazzini, Fabiola Olivieri, Maria Giulia Bacalini, Rita Ostan, Daniela Mari, Giuseppe Passarino, Daniela Monti, Anna Rita Bonfigli, Massimo Boemi, Antonio Ceriello, Stefano Genovese, Federica Sevini, Donata Luiselli, Paolo Tieri, Miriam Capri, Stefano Salvioli, Jan Vijg, Yousin Suh, Massimo Delledonne, Roberto Testa, Claudio Franceschi

**Affiliations:** ^1^ DIMES - Department of Experimental, Diagnostic and Specialty Medicine, University of Bologna, Bologna, 40126 Italy; ^2^ C.I.G. Interdepartmental Center “L. Galvani”, University of Bologna, Bologna, Italy; ^3^ CRBA - Applied Biomedical Research Center, S. Orsola-Malpighi Polyclinic, Bologna, 40138 Italy; ^4^ Department of Biological, Geological and Environmental Sciences, Laboratory of Molecular Anthropology & Centre for Genome Biology, University of Bologna, Bologna 40126, Italy; ^5^ Department of Clinical and Molecular Sciences, Università Politecnica delle Marche, Ancona, Italy; ^6^ Center of Clinical Pathology and Innovative Therapy, Italian National Research Center on Aging INRCA-IRCCS, Ancona, Italy; ^7^ Geriatric Unit IRCCS Ca' Granda Foundation Maggiore Policlinico Hospital Hospital and Department of Clinical Sciences and Community Health, University of Milano, Italy; ^8^ Department of Cell Biology, University of Calabria, Rende, Italy; ^9^ Department of Experimental and Clinical Biomedical Sciences, University of Florence, Florence, Italy; ^10^ Metabolic Diseases and Diabetology Unit, IRCCS-INRCA, Ancona, Italy; ^11^ Institut d'Investigacions Biomèdiques August Pi i Sunyer (IDIBAPS), Barcelona, Spain; ^12^ Centro de Investigación Biomédica en Red de Diabetes y Enfermedades Metabólicas Asociadas (CIBERDEM), Barcelona, Spain; ^13^ Department of Cardiovascular and Metabolic Diseases, IRCCS Gruppo Multimedica Sesto San Giovanni (MI), Italy; ^14^ IAC-CNR Istituto per le Applicazioni del Calcolo, Consiglio Nazionale delle Ricerche, Rome, Italy; ^15^ Department of Genetics, Albert Einstein College of Medicine, Bronx, NY 10461, USA; ^16^ Department of Medicine, Albert Einstein College of Medicine, Bronx, NY 10461, USA; ^17^ Institute for Aging Research, Diabetes Research and Training Center, Albert Einstein College of Medicine, Bronx, NY 10461, USA; ^18^ Institute of Aging Research, Guangdong Medical College, Dongguan 523808, China; ^19^ Personal Genomics SRL, Strada le Grazie 15, 37133 Verona - Italy; ^20^ Functional Genomics Center, Dept. of Biotechnologies, University of Verona, Strada le Grazie 15, 37133 Verona - Italy; ^21^ Experimental Models in Clinical Pathology, IRCCS-INRCA, Ancona, Italy

**Keywords:** Type 2 diabetes, TCF7L2, centenarians, extreme phenotypes, age-related diseases

## Abstract

Genetic association studies of age-related, chronic human diseases often suffer from a lack of power to detect modest effects. Here we propose an alternative approach of including healthy centenarians as a more homogeneous and extreme control group. As a proof of principle we focused on type 2 diabetes (T2D) and assessed allelic/genotypic associations of 31 SNPs associated with T2D, diabetes complications and metabolic diseases and SNPs of genes relevant for telomere stability and age-related diseases. We hypothesized that the frequencies of risk variants are inversely correlated with decreasing health and longevity. We performed association analyses comparing diabetic patients and non-diabetic controls followed by association analyses with extreme phenotypic groups (T2D patients with complications and centenarians). Results drew attention to rs7903146 (TCF7L2 gene) that showed a constant increase in the frequencies of risk genotype (TT) from centenarians to diabetic patients who developed macro-complications and the strongest genotypic association was detected when diabetic patients were compared to centenarians (p_value = 9.066*10^−7^). We conclude that robust and biologically relevant associations can be obtained when extreme phenotypes, even with a small sample size, are compared.

## INTRODUCTION

In the last ten years the scientific community has devoted a consistent effort to identify the genetic basis of the most common age-related diseases, as they represent one of the most important public health and socio-economical burden all over the world and particularly in Western Countries. This challenge was mainly faced up by genome wide association studies (GWASs) based on microarray technology that allows the simultaneous analyses of hundred thousands of single nucleotide polymorphisms (SNPs), within the framework of the “common variant common disease” theory [1]. So far, more than 1,000 published GWASs reported significant associations of ~4,000 SNPs for more than 200 traits/diseases [2]. GWASs of age-related, chronic human diseases often suffer from a lack of power to detect modest effects, which can to some extent explain why the identified genetic effects comprise only a small fraction of the estimated trait heritability. These limitations can be overcome simply by ever increasing sample size in order to achieve the necessary statistical power to detect variants with small effects, which is not always feasible. Moreover, in most cases the biological role/relevance of the genetic variants emerged from GWASs is still unclear. In past, several strategies were proposed to increase detection power without affecting the cohort size, such as the use of genetically isolated populations characterized by a reduced genetic diversity that facilitate the discovery of relevant loci for complex diseases [3,4]. To date this approach has not been successful to boost the discovery of relevant associations with complex traits [5]. Thus, there is an urgent need for an effective strategy to obtain biological insights from the genetic knowledge derived from GWASs that can be translated into clinical benefits.

The aim of this study was to test the hypothesis that GWASs sensitivity can be boosted by including extreme phenotypic groups. As a proof of principle study, we considered one of the major age-related diseases, *i.e.* type 2 diabetes (T2D). We applied a candidate gene approach to assess associations of a limited set of SNPs (31) in or nearby genes relevant for T2D, diabetes complications, metabolic diseases, telomere stability and age-related diseases. The extreme phenotypic groups we focused on are as follows: i) *CENTENARIANS* who reached the extreme limit of human life, escaping or largely postponing the major age-related diseases, including T2D, and who can be considered a paradigm of healthy aging [6]. In particular, a major characteristic of centenarians is their extraordinarily well preserved glucose metabolism. Aging is frequently associated with impaired glucose metabolism related to a raise in insulin resistance (IR), not fully compensated by a sufficient β-cell function. Such age-related metabolic changes are key risk factors for T2D and are associated with a variety of intermediate phenotypes (hypertension, atherosclerosis, obesity) strongly affecting morbidity, disability and mortality among elderly. In a pioneering study, Paolisso et al. [7], using an oral glucose tolerance test and euglycemic glucose clamp, showed that centenarians had a 2-h plasma glucose concentration that was lower than that of aged subjects but not different from adults, and an insulin-mediated glucose uptake higher than that reported for aged subjects but not different from that found in adults. In a subsequent study, involving a large cohort of individuals (age range: 28-111 years) carefully selected for health status, we showed that the age-related trajectories of IR and β-cell function increase with age (the increase of β-cell function is necessary to compensate for the raise in IR). However, beyond 85-90 years of age, people with a lower degree of IR and with a lower β-cell function emerge, indicating that in very old people lower IR does not require a compensatory increment in β-cell function, thus allowing to preserve endocrine pancreas secretion and to prevent the development of T2D [8]. On the whole, these results point towards IR and β-cell function as phenotypes under strong selective forces during aging, supporting the hypothesis that effective peripheral glucose disposal is pivotal in determining longevity. These observations are confirmed by studies from our and other groups showing that centenarians offspring and nonagenarian siblings have a better health status than subjects of the same cohort, without the parental extreme longevity [9], and a reduced risk of diabetes and an enhanced insulin sensitivity [10-14]. ii) *PATIENTS WITH T2D PLUS ONE OR MORE COMPLICATIONS*. The T2D patients included in the present study have been fully characterized from a phenotypical (clinical, biochemical, pharmacological, among others) point of view, and have been extensively studied regarding a variety of genetic [15-18] and non-genetic factors. In particular, we showed that T2D patients undergo accelerated aging [19] and that *IL-10* polymorphisms [20], telomere length in peripheral blood cells [21] and mitochondrial DNA variants [22] are able to distinguish between patients with and without complications. Thus, the presence of one or more micro- and macro-vascular complications not only represents the most severe and extreme phenotype of T2D, of relevance from a clinical and therapeutic point of view, but also has a biological counterpart which is still largely unknown. In this study, T2D patients have been compared with age-, gender- and geography-matched control group.

Here we considered a limited set of SNPs belonging to four classes of genetic variants: i) SNPs previously found associated with T2D by GWASs; ii) variants of genes involved in vascular pathology potentially involved in the development of T2D complications; iii) SNPs previously associated with metabolic diseases, including T2D; and iv) SNPs of genes relevant for telomere stability and involved in age-related diseases, including T2D complications.

## RESULTS

**Figure 1:**
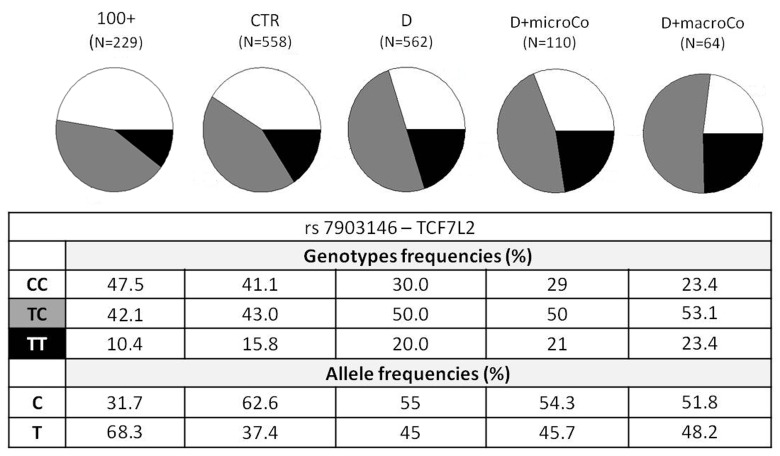
*TCF7L2* rs7903146 genotypic and allelic frequencies among the different groups analyzed *TCF7L2* rs7903146 genotypic and allelic frequencies were reported according to the decreasing of health/longevity and the increasing of T2D severity (from left to right).

In this study we analyzed the following cohorts described in depth in Methods: the whole cohort of diabetic patients (D), diabetic patients with complications (D+Co), diabetic patients who developed micro-vascular complications (D+microCo), diabetic patients who developed macro-vascular complications (D+macroCo), non diabetic controls matched for age, gender and geographical origin with diabetic patients (CTR) and centenarians (100+). These samples were tested for 31 SNPs, 22 of which passed the quality check. Results from association analyses are reported in Supplemental Materials (S1, S2, S3, S4). The most significant results involve *TCF7L2* and *ADIPOQ* gene variants, which are reported in Table 1 and summarized in Figure 1. The comparisons that have been performed to test the effectiveness of our approach are reported in the following five sections.

**Table 1:** Allelic and genotypic association analyses. Samples considered were diabetic patients (N=562), non diabetic controls (N=558), diabetic individuals with complications (N=241), centenarians (N=229), diabetic patients with macro-vascular complications (N=64) and diabetic patients with micro-vascular complications (N=110).

	Cohorts	Gene	SNP (ID)	Allele/Genotype	Frequency (%)		p_value	OR (95% CI)	Model
					1	2			
Allelic Association	D (1) vs 100+ (2)	*ADIPOQ*	rs266729	G	24.6	31.6	5.89*10^−3^	0.71 (0.55-0.91)	/
	D (1) vs CTR (2)	*TCF7L2*	rs7903146	T	45	37	3.1*10^−4^	1.37 (1.16-1.63)	/
	D+Co (1) vs CTR (2)	*TCF7L2*	rs7903146	T	46.1	37	1.66*10^−3^	1.43 (1.14-1.79)	/
	D (1) vs 100+ (2)	*TCF7L2*	rs7903146	T	44.8	31.4	1.34*10^−6^	1.77 (1.40-2.24)	/
	D+Co (1) vs 100+ (2)	TCF7L2	rs7903146	T	45	31.4	1.06*10^−6^	1.78 (1.41-2.25)	/
	D+macroCo (1) vs 100+ (2)	TCF7L2	rs7903146	T	50	31.4	1.13*10^−4^	2.177 (1.46-3.24)	/
	D+microCo (1) vs 100+ (2)	*TCF7L2*	rs7903146	T	46	31.4	2.64*10^−4^	1.85 (1.33-2.57)	/
**Genotypic association**	D (1) vs 100+ (2)	*ADIPOQ*	rs266729	G/C	36.6	50.7	4.19*10^−4^	0.56 (0.41-0.77)	Over dominant
	D (1) vs CTR (2)	*TCF7L2*	rs7903146	TC/TT	58.9	70.0	1.41*10^−4^	1.63 (1.26-2.09)	Dominant
	D+Co (1) vs CTR (2)	*TCF7L2*	rs7903146	TC/TT	58.8	71.9	5.96*10^−4^	1.79 (1.28-2.52)	Dominant
	D (1) vs 100+ (2)	*TCF7L2*	rs7903146	CC	30	47.5	9.07*10^−7^	1.78 (1.41-2.26)	Log-additive
				TC	50	42.1			
				TT	20	10.4			
	D+Co (1) vs 100+ (2)	*TCF7L2*	rs7903146	CC	28.1	47.5	5.47*10^−6^	1.89 (1.43-2.50)	Log-additive
				TC	51.1	42.1			
				TT	20.8	10.4			
	D+macroCo (1) vs 100+ (2)	*TCF7L2*	rs7903146	CC	23.4	47.5	1.54*10^−4^	2.18 (1.45 −3.29)	Log-additive
				TC	53.1	42.1			
				TT	23.4	10.4			
	D+microCo (1) vs 100+ (2)	*TCF7L2*	rs7903146	CC	29.1	47.5	3.30*10^−4^	1.84 (1.31 −2.58)	Log-additive
				TC	50.0	42.1			
				TT	20.9	10.4			

### All diabetic patients (D) *vs* non diabetic controls (CTR) (classical approach of genetic association studies)

Allelic and genotypic association analyses were performed on all diabetic patients *vs* the classical control group. Only *TCF7L2* rs7903146 T-allele was associated with T2D with an odds ratio (OR) of 1.372 per risk allele (95% CI 1.155-1.629) (Table 1). Genotypic association of the *TCF7L2* rs7903146 was significantly detected (p_value = 1.415*10^−4^, OR = 1.63 (95% CI 1.26-2.09)) under a dominant model (Table 1).

### All diabetic patients (D) *vs* centenarians (100+)

Allelic and genotypic association analyses were performed on all diabetic patients *vs* centenarians. Two nominal significant differences in the allelic frequencies of diabetic patients and centenarians were observed in the *ADIPOQ* rs266729 (OR = 0.706 (95% CI 0.551 - 0.905), p_value = 5.89*10^−3^) and in *TCF7L2* rs7903146. In this comparison the *TCF7L2* rs7903146 was highly associated with T2D with an OR = 1.775 per risk allele (95% CI 1.404-2.243, p_value = 1.35*10^−6^) (Table 1). *TCF7L2* association was supported by genotypic association analysis. Indeed, significant differences in genotypic frequencies for rs7903146 were reported (OR = 1.78 (95% CI 1.41-2.26), p_value = 9.066*10^−7^) (Table 1). Genotypic association was found also for rs266729 in *ADIPOQ* gene (p_value = 4.19*10^−4^).

**Diabetic patients with complications (D+Co) *vs* non diabetic controls (CTR)** Allelic and genotypic association analyses were performed on diabetic patients with at least one complication *vs* non diabetic controls. Only *TCF7L2* rs7903146 showed an allelic association with T2D susceptibility with an OR = 1.434 (95% CI 1.145-1.795, p_value = 1.66*10^−3^). No allelic associations were detected for the other SNPs genotyped (Supplementary Table 2). Genotypic association was observed for the same SNP with an OR = 1.79 (95% CI 1.28-2.52, p_value = 5.96*10^−4^) (Table 1).

**Table 2:** Diabetic patients and non diabetic controls collected by the Diabetology Unit in Ancona: samples description

		All samples		Males		Females	
	Variables	Diabetic Patients (N=562)	Controls (N=558)	Diabetic Patients (N=305)	Controls (N=214)	Diabetic Patients (N=257)	Controls (N=344)
**Traits**	Age ± STD	65.76± 8.11	58.11 ± 12.40	64.93 ± 8.42	58.35 ± 11.88	66.75 ± 7.64	57.96 ±12.73
	Sex (M/F)	306/258	213/345	…	…	…	…
	BMI ± STD	28.76 ±4.62	27.14 ± 4.46	28.24 ± 4.15	27.29±3.51	29.40 ± 5.07	27.04 ±5.00
**Complications**	Retinopathy (%)	27.22 %	…	27.21 %	…	27.24 %	…
	Somatic Neuropathy (%)	17.79 %	…	21.64 %	…	13.23 %	…
	Renal failure (%)	3.56 %	…	4.92 %	…	1.94 %	…
	Nephropathy (%)	12.81 %	…	16.06 %	…	8.95 %	…
	Ischemic heart disease (%)	17.08 %	…	20.33 %	…	13.23 %	…
	Acute myocardial infarction (%)	9.07 %	…	13.11 %	…	4.28 %	…
	Ictus (%)	6.76 %	…	6.88 %	…	6.61%	…

### D+Co*vs* 100+, D+macroCo *vs* 100+, and D+microCo *vs* 100+ (comparison between extreme phenotypes)

Genotypic association analyses were performed on diabetic patients with at least one complication *vs* centenarians as a non-diabetes control group. When we compared D+Co *vs* 100+, D+macroCo *vs* 100+ and D+microCo *vs* 100+, the *TCF7L2* rs7903146 resulted associated with T2D complicated patients with an OR = 1.89 (95% CI 1.43-2.50, p_value = 5.473*10^−6^), 2.18 (95% CI 1.45 -3.29, p value = 1.538*10^−4^) and 1.84 (95% CI 1.31 - 2.58, p_value = 2.68*10^−4^), respectively (Table 1). In Figure 1 allele and genotype frequencies for rs7903146 were reported for each group considered.

### TCF7L2 interactome analysis

**Figure 2:**
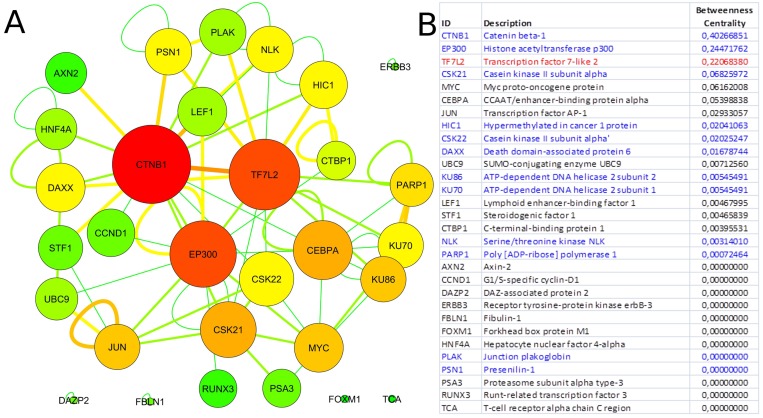
*Tcf7l2* interactome Graph of the *Tcf7l2* interactome (**A**) and list of its elements (**B**). The most central elements in terms of betweenness centrality and node degree [85,89] are *Ctnb1, Tfc7l2* (as expected) and *Ep300*. Non-connected nodes: *TCF7L2* gene-listed gene interactions only, and no PPIs, are reported. (**A**) Node color code: from red to green, from highest to lowest values of betweeness centrality; interaction link color code: from red to green, from higher to lower number of experimental evidences. (**B**) Direct physical interactors are in blue, other types of interaction, such as colocalization and physical association are in black.

**Figure 3:**
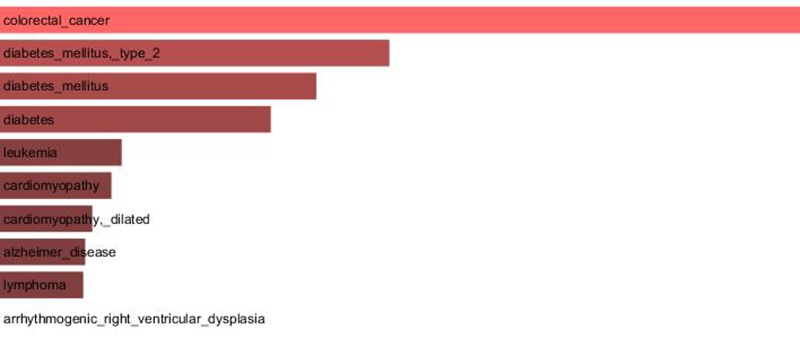
Major OMIM diseases linked to the Tcf7l2 interactome OMIM enrichment analysis of the Tcf7l2 interactome was performed using the Enrichr web service (http://amp.pharm.mssm.edu/Enrichr/) and the first ten OMIM diseases significantly linked to the interactome (“Enrichr combined score”) are reported.

Since our results confirmed a core role of *TCF7L2* in T2D pathogenesis, consistent with its implicated role in T2D [23,24], an interactome analysis was performed. Tcf7l2 is a 619 AA-long transcription factor that participates in the Wnt signaling pathway and modulates *MYC* expression by binding to its promoter in a sequence-specific manner. The most central elements of the Tcf7l2 interactome are Ctbn1 (ranking 1^st^) and Ep300, and, on a second order of importance, Csk21, Myc and Cebpa (Figure 2A). Among the whole Tcf7l2 interactome (including co-localization and physical association) with 30 elements (Figure 2), there is evidence of direct physical interactions (experimentally validated protein-protein interaction) of Tcf7l2 (Figure 2B, red line) with 12 other proteins (Figure 2B, blue lines). Listing OMIM enrichment analysis of the Tcf7l2 interactome was performed and the first ten OMIM diseases significantly connected to the interactome are reported in Figure 3.

## DISCUSSION

In the last few years GWASs provided a great amount of data regarding genetic risk factors for major age-related diseases. Most variants identified so far confer relatively small increments in risk, and explain only a small proportion of the estimated trait heritability. Increasing sample size has been suggested as an answer to the missing heritability to increase sensitivity to detect variants with smaller effects. As an alternative for dramatically increasing cohort size, we tested a strategy that involves extreme phenotypes replicating a limited set of SNPs emerged and validated in GWASs. Individuals that are at the extreme ends of a trait distribution have been proposed for identifying variants that are rare but not private and that have modest to high effect sizes.

We hypothesized that the frequencies of GWASs risk variants are inversely correlated with increasing health and longevity, and extreme phenotypic differences between cases and controls would allow detection of the genetic variants with the most relevant biological and clinical value at relatively small sample size. For an extreme-trait approach, accurate phenotyping will be of vital importance. Central to our approach is the “control” group. The proper “normal” controls, especially in relation to common age-related diseases, are difficult to define, especially when large numbers of “healthy” subjects are collected from different regions, characterized by peculiar lifestyle habits, population genetics, among others. To circumvent these difficulties, we propose to use centenarians as an additional, more informative and defined control group for studies on the genetic determinants of age-related diseases, and particularly to establish the biological and clinical relevance of the genetic variants emerged in GWASs. Indeed, this group can be considered as a “super-control”, as most centenarians achieved their remarkable age avoiding or largely postponing major age-related diseases, and thus, at variance with much younger controls, we can be sure that they never developed such diseases. We reasoned that if a given genetic variant emerged from GWASs and considered a statistically significant but “weak” risk factor for the disease of interest, is present at similar frequency in centenarians and in patients, its biological relevance is reasonably negligible. On the contrary, if a given genetic variant considered a risk factor for an age-associated disease has a frequency much higher in patients than in centenarians, we can reasonably argue that this variant is a strong candidate to play a consistent biological role in the pathogenesis of the disease. We also assumed that centenarians, as the best model of healthy aging in humans [6], are a control group less blurred and undefined than the usually employed “age-matched controls”. Moreover, in order to properly take into account the above-mentioned largely neglected clinical heterogeneity/severity of age-related diseases in studies on their genetic determinants, we focused on T2D and more specifically on two main groups of patients, *i.e*. those who did not develop complications and can enjoy a relatively long and acceptable quality of life, and those who developed one or more (micro- and macro-vascular) complications with a poorer quality of life. Accordingly, this approach allowed us to compare *extreme phenotypes, i.e.* centenarians on one side and T2D patients with severe complications on the other, thus maximizing possible genetic differences among patients and controls.

Using this methodological approach on a total of 1,349 individuals we tested 31 SNPs in or nearby 16 different genes that were previously strongly associated with T2D and metabolic diseases and that could be considered good candidate to play a role in T2D complications, on the basis of other large genetic studies.

Our results indicate that most of the SNPs analyzed were not significantly associated with T2D, even when extreme groups of cases and controls were used. This somewhat unexpected result could be due to a lack of biological relevance in spite of their previously strong association signal in GWASs. Indeed, a major drawback of GWASs is the observation of many significant association signals from loci of weak effect. Therefore, we have to consider the possibility that such SNPs, despite having reached statistical significance in large GWASs, could have a weak biological relevance. Indeed, the presence of risk alleles at the same frequency in T2D patients with or without complications and in centenarians suggests that *per se* they do not represent a strong biological risk, being compatible with exceptional longevity, and that they likely necessitate to interact (epistatic effects) with (many) other risk alleles and/or specific environmental conditions to give a certain phenotype. Further studies, possibly in more than one population, are needed to test this possibility.

The two SNPs that reached statistical significance in our study were *ADIPOQ* rs266729 and *TCF7L2* rs7903146, the last one being the strongest. *ADIPOQ* encodes for adiponectin, the most abundant adipokine in human plasma. Low adiponectin levels are associated to IR and T2D [26-28] and the chromosome locus 3q27, where *ADIPOQ* is located, is linked to metabolic syndrome and T2D [29]. Several studies have investigated the association between *ADIPOQ* polymorphisms and T2D, but their results are controversial, probably due to differences in the size and in the composition of the analyzed cohorts [30]. To this regard it is interesting to note that the statistical significance of *ADIPOQ* rs266729, a polymorphism associated to decreased adiponectin levels, emerged only when T2D patients were compared with centenarians. Regarding *TCF7L2* rs7903146, localizedin an intronicregion of the gene, the most interesting findings were that the risk allele T and the genotype TT showed an increasing statistical significance, from modest genotypic association with the disease when all T2D patients (D) were compared with the age-matched controls (CTR) (OR=1.63; 95% CI 1.26-2.09) to intermediate values (OR=1.79; 95% CI 1.28-2.52) when T2D patients with complications (D+Co) were compared with CTR. These OR values perfectly overlap OR previously described in literature [31] in GWASs that analyzed thousands of samples. Finally, the OR and p_values showed the most significant results when centenarians were compared with T2D patients, and a further increase in OR was observed when centenarians and patients with complications, the extreme phenotypes, were compared (D *vs* 100+*:* OR=1.78 (95% CI 1.41-2.26), p_value=9.066*10^−7^; D+Co *vs* 100+*:* OR=1.89 (95% CI 1.43-2.50), p_value=5.473*10^−6^). It is important to note that the reverse is true for the C allele and the CC genotype. Notably, centenarians show not only the lowest frequency of T allele and TT genotype, but also the highest frequency of C allele and CC genotype, which emerges as a robust protective longevity variant. These data also suggest that *TCF7L2* rs7903146 is a robust genetic risk variant and indicate that further analyses based on the use of centenarians as super-controls, possibly at genome wide level, are worthwhile to pursue in T2D study, and likely in other age-related diseases. TT genotype frequency was found to be higher not only in diabetic patients but also in patients with macro-vascular complications (Figure 1), reaching a frequency of 23.4%. These results demonstrate an important role of rs7903146 not only in T2D susceptibility but also in susceptibility to develop macro-vascular complications. Different studies investigated the relationship between macro-vascular complications, cardiovascular diseases and diabetes but the molecular mechanisms are still unknown [32-35].

The *TCF7L2* polymorphism rs 7903146 was one of the first tobe related to diabetes [36]and subsequently its association has been confirmed in several studies, placing it among the most reproducible markers of T2D [37-40]. Recent studies have shown that this SNP leads to conformational changes inchromatin structure according to the allele type. In particular the risk allele (T) inducesan open conformation of the chromatin, resulting in an increased transcriptional activityand *TCF7L2* mRNA levels [39,41,42]. It has been demonstrated that the increase of expression of *TCF7L2* leads to a progressive reduction of glucose to lerance *in vivo* [43].

In order to gain information about the biological relevance of *TCF7L2* gene, we performed an interactome analysis, which identified a complex network of interactions with Ctnb1 and Ep300 as the central elements. Ctnb1 is a Beta-catenin (adherens junction protein), critical for the establishment and maintenance of epithelial layers, and a key downstream component of the canonical Wnt signaling pathway. Ep300 is a histone acetyltransferase and regulates transcription via chromatin remodeling. Chromatin remodeling and epigenetic mechanisms seem to play a fundamental role in susceptibility to diabetes and related complications [44-47] even if mechanisms are not completely understood and further studies should be designed to clarify them. The *in silico* analysis suggests that Tcf7l2 interactome is involved in a variety of age-related pathologies, such as Alzheimer's disease, cardiovascular diseases and colorectal cancer [48-52], all sharing vascular alterations, as a further support to the biological relevance of this gene and to the emerging idea that the same gene can play a major role in apparently different diseases [53].

On the whole, our data on *TCF7L2* rs7903146 indicate that the frequency of T2D risk genotypes decreases according to the severity of the disease phenotype, supporting the assumption that centenarians represent a powerful and informative control group in association studies on T2D, a pathology characterized by significant clinical heterogeneity. Different authors have emphasized the problem of phenotypical heterogeneity of cohorts, which can seriously affect the ability todetect genetic associations [54]. Our data suggest that the use of relatively small numbers of cases and controls with extreme phenotypes (thus reducing sample heterogeneity) in genetic association studies of age-related diseases can be successful in detecting significant associations as suggested by other authors [55].

## METHODS

### Samples

A total of 1,349 individuals from Northern/Central Italy, including 562 T2D patients (D) (mean age: 65.76 ± 8.11) and 558 unrelated age-, gender- and geographically-matched controls (CTR) (mean age: 58.11 ± 12.40), and 229 centenarians (100+) (mean age: 105.0 ± 2.9) have been considered in this study. D and CTR subjects were collected by the Diabetology Unit, INRCA (National Institute on Health and Science on Aging) in Ancona (Italy). T2D diagnosis was made according to the American Diabetes Association Criteria (www.diabetes.org) All patients were fully characterized from a clinical point of view and a large number of biochemical/endocronological parameters were measured. To avoid population stratification effects, only patients and controls with at least two generations of maternal ancestry from the Marche region (Central Italy) were included in this study. CTR subjects were carefully assessed and fully characterized according to the same protocol used for T2D patients, and a detailed clinical history was recorded in order to exclude the presence of T2D and of any other overt illness. Within the group of diabetic patients, N = 241 were affected by complications (D+Co), and in particular N = 110 had only micro-vascular (D+microCo) and N = 64 had only macro-vascular complications (D+macroCo). The main phenotypical and clinical characteristics of D and CTR are summarized in Table 2.

The presence/absence of microvascular and macrovascular diabetic complications was assessed according to the following criteria: i) *microvascular*: diabetic retinopathy by fundoscopy through dilated pupils and/or fluorescence angiography; incipient nephropathy, defined by an excessive urinary albumin excretion (>30 mg/24 h) and a normal creatinine clearance; renal failure, detected as an estimated glomerular filtration rate >60 mL/min per 1.73 m2; neuropathy established by electromyography; ii) *macrovascular*: ictus by clinical history; ischemic heart disease and acute myocardial infarction by clinical history and by resting electrocardiogram; peripheral vascular disease by clinical history and, for lower limbs, by ankle-brachial index. Hypertension was defined as a systolic blood pressure >140 mmHg and/or a diastolic blood pressure >90 mmHg. The values were measured while the subjects were sitting and confirmed at least three times. Overnight fasting venous blood samples from all subjects were collected from 8:00 to 9:00 a.m. Blood concentrations for HDL cholesterol, triglycerides, HbA1c, fasting insulin, fibrinogen, high-sensitivity C reactive protein (hsCRP), creatinine, urea nitrogen, and white blood cells count were measured by standard procedures.

All centenarians were born in Italy between the 1900 and 1908. Trained physicians and nursing staff collected demographic and lifestyle data, anthropometric measurements, functional, cognitive and health status, clinical anamnesis, and details on drug use, as previously described [9].

### SNPs selection

A total of 31 SNPs mapping within and nearby 16 different genes (Table S5) were genotyped in the 1,349 individuals included in the study. The SNPs were selected according to the following criteria: *i) SNPs reported to be risk* factor for T2D [56-62]such as Transcription factor 7-like 2 (*TCF7L2*), Insulin-like growth factor 2 mRNA-binding proteins (*IGF2BP*), Potassium inwardly-rectifying channel, subfamily J, member 11 (*KCNJ11*), Potassium voltage-gated channel KQT-like sub-family, member 1 (*KCNQ1*); *ii)SNPs of candidate genes for the development of T2D complications*, such as Catalase (*CAT*), erythropoietin (*EPO*) [61], hypoxia-inducible factor 1 α subunit (*HIF1A*) [63] and *DDAH1*. Interestingly, significant association of *CAT* and *SOD* polymorphisms with T2D were recently reported [64]. Genetic variations in the *DDAH1* and *DDAH2* genes are significantly associated with serum Asymmetric DiMethylArginine (ADMA) levels in T2D patients [65]. Moreover, it was reported that *DDAH* gene polymorphisms play a central role in determining ADMA in diabetic renal impairment [66]. *iii)SNPs of genes reported to be involved in metabolic diseases, including T2D,* such as *ADIPOQ* and *IRS1* encoding for adiponectin and insulin receptor substrate 1 respectively, and *FTO*, a major gene for obesity susceptibility [62-65]. Adiponectin is an adipocyte-produced protein involved in regulating glucose, lipid and energy metabolism, and *ADIPOQ* polymorphisms were previously associated with T2D in Caucasian and non-Caucasian populations [59,71,72]. *IRS1* polymorphisms have been associated with risk of T2D and adiposity in GWASs. Recently, it was reported that *IRS1* rs2943641 interacts with carbohydrate and fat intakes in incident T2D in a sex-specific manner [73]; *iv)SNPs of genes relevant for aging and age-related diseases*, such as *hTERT* and *TERC*, involved intelomere stability/attrition and in T2D complications [21,74,75].

### SNPs genotyping

DNA was extracted from whole blood (QIAmp 96 DNA Blood kit, QIAGEN). Genotyping analysis was performed by using SEQUENOM MassArray iPLEX technology, following the manufacturer's instructions and as previously described [76,77]. Genotype calls were analyzed by using SEQUENOM Typer 4.0 software and the individual spectrograms were checked in order to evaluate the presence of calling errors.

### SNP analysis and quality control

Four SNPs (rs16889462, rs4880, rs8047395 and rs10434) that did not satisfy the Hardy-Weinberg equilibrium (HWE) in the control group and five SNPs (rs669173, rs2853669, rs3025021, rs13266634 and rs7901695) that showed 25% missing call rates were excluded from the analysis (Table S5). Diabetic patients were also analyzed after stratifications for the type of complications (micro and macro-vascular).

Only SNPs with call rates higher than 95%, without significant deviation from HWE in controls and with minor allele frequency (MAF) exceeding 5% were retained for the association analysis. Genotypic analysis was performed using R 2.15.2 and SNPassoc package (http://www.r-project.org) [78] that implements binary logistic regression methods under five different genetic models. Allelic association analyses were performed using PLINK 1.07, an open-source whole genome association analysis toolset (http://pngu.mgh.harvard.edu/purcell/plink/) [71].

### Interactome analysis

Protein-protein interaction (PPI) data have been retrieved from the Agile Protein Interactions Database APID [80] accessed through the dedicated plugin APID2NET [81] and from the metadatabase InnateDB [82] and then integrated to form the Tcf7l2 interactome (i.e. the set of molecular interactions related to Tcf7l2) and analyzed with the network analysis platform Cytoscape [83,84]*.* Topological measures such as betweenness centrality and node degree are used to rank the importance of the interactome elements [85,86]. The betweenness centrality of a node reflects the amount of control that this node exerts over the interactions of other nodes in the network. This measure favors nodes that join communities (dense subnetworks), rather than nodes that lie inside a community [87,88]. OMIM diseases overrepresentation analyses and ranking have been performed using Enrichr (http://amp.pharm.mssm.edu/Enrichr).

## SUPPLEMENTAL DATA


