# Supplementary material for: Centenarians as super-controls to assess the biological relevance of genetic risk factors for common age-related diseases: A proof of principle on type 2 diabetes
Source: Aging (Albany NY). 2013 May 31;5(5):373–85. doi: 10.18632/aging.100562 (PMC3701112; doi:10.18632/aging.100562)
Supplement: Supplementary file 1 [file aging-05-373-s001.doc]

**SUPPLEMENTAL DATA**

| **TABLE S1.** Allelic association: D *vs* CTR and D *vs* 100+ | | | | | | | | | | | |
| --- | --- | --- | --- | --- | --- | --- | --- | --- | --- | --- | --- |
|  | | **D *vs* CTR** | | | | | **D *vs* 100+** | | | | |
| Gene | SNPs ID | Allele | Frequency | | p-value | OR (95% CI) | Allele | Frequency | | P_value | OR (95% CI) |
|  |  |  | D CTR  N=562 N=558 | |  |  |  | D  N=562 | 100+  N=229 |  |  |
| *DDAH1* | rs7521189  rs13373844 | G  C | 0.4532  0.3373 | 0.4694  0.3203 | 0.4439  0.396 | 0.9369 (0.793-1.107)  1.08 (0.9041-1.29) | G  C | 0.4529  0.339 | 0.4933  0.298 | 0.1472  0.1182 | 0.850 (0.682-1.06)  1.208 (0.953-1.532) |
| *IRS1* | rs2943641 | T | 0.3989 | 0.3761 | 0.2722 | 1.101 (0.9274-1.307) | T | 0.3984 | 0.3683 | 0.2713 | 1.136 (0.9053-1.425) |
| *TERC* | rs12696304 | G | 0.2746 | 0.278 | 0.8584 | 0.9832 (0.8165-1.184) | G | 0.2748 | 0.2227 | 0.03518 | 1.322 (1.019-1.715) |
| *IGF2BP* | rs4402960  rs1470579 | T  C | 0.3621  0.3527 | 0.3203  0.3028 | 0.0387  0.01395 | 1.205 (1.01-1.437)  1.254 (1.047-1.503) | C  T | 0.3527  0.3621 | 0.2986  0.3054 | 0.04254  0.0349 | 1.28 (1.008-1.626)  1.29 (1.018-1.635) |
| *ADIPOQ* | rs266729  rs1063539 | G  C | 0.2455  0.1082 | 0.2356  0.1132 | 0.5843  0.7195 | 1.056 (0.8693-1.282)  0.9508 (0.7219-1.252) | G  C | 0.2464  0.1082 | 0.3164  0.1527 | 5.896*10^-4^  0.01582 | 0.7063 (0.5511-0.9052)  0.6724 (0.4864-0.9296) |
| *hTERT* | rs2736098  rs2735940  rs2736109  rs401681 | A  C  A  T | 0.2952  0.4088  0.4099  0.4203 | 0.3056  0.4161  0.4304  0.4143 | 0.6084  0.7299  0.3328  0.7745 | 0.9516 (0.787-1.151)  0.9706 (0.8196- 1.15)  0.9194 (0.7755-1.09)  1.025 (0.8659-1.213) | C  A  A  C | 0.41  0.4188  0.4113  0.2949 | 0.4051  0.4276  0.4182  0.2977 | 0.8604  0.7506  0.8041  0.9154 | 1.021 (0.8136-1.28)  0.9645 (0.7716-1.205)  0.9719 (0.7763-1.217)  0.9868 (0.771-1.262) |
| *EPO* | rs1617640  rs507392  rs551238 | G  C  C | 0.3229  0.3312  0.3246 | 0.3423  0.3512  0.3418 | 0.33  0.3262  0.3914 | 0.9161 (0.768-1.093)  0.9147 (0.7656-1.093)  0.9256 (0.7755-1.105) | C  G  C | 0.3312  0.3234  0.3245 | 0.3226  0.2707  0.32 | 0.7483  0.04852  0.8621 | 1.04 (0.8195-1.319)  1.288 (1.001-1.656)  1.021 (0.8073-1.291) |
| ***TCF7L2*** | **rs7903146** | **T** | **0.45** | **0.3736** | **3.1*10^-4^** | **1.372 (1.155-1.629)** | **T** | **0.4488** | **0.3145** | **1.35*10^-6^** | **1.775 (1.404-2.243)** |
| *CAT* | rs1001179 | A | 0.2158 | 0.2116 | 0.8155 | 1.025 (0.8328-1.262) | T | 0.05535 | 0.04167 | 0.2753 | 1.348 (0.787-2.308) |
| *KCNJ11* | rs5215 | C | 0.3565 | 0.3348 | 0.2846 | 1.1 (0.9234-1.311) | C | 0.3584 | 0.3496 | 0.7398 | 1.04 (0.8267-1.307) |
| *KCNQ1* | rs2237892 | T | 0.05495 | 0.03874 | 0.07062 | 1.443 (0.9678-2.151) | A | 0.2147 | 0.1895 | 0.2754 | 1.169 (0.8826-1.549) |
| *HIF-1α* | rs11549465 | T | 0.1452 | 0.1505 | 0.7252 | 0.9589 (0.7587-1.212) | T | 0.1444 | 0.125 | 0.3162 | 1.181 (0.8525-1.637) |
| *FTO* | rs8050136  rs9939609 | A  A | 0.4731  0.4683 | 0.4387  0.4315 | 0.1041  0.08209 | 1.149 (0.9719-1.357)  1.16 (0.9812-1.372) | A  G | 0.4738  0.469 | 0.4951  0.4409 | 0.4626  0.3184 | 0.9183 (0.7314-1.153)  1.12 (0.8965-1.399) |

**Table S1**. Allelic association comparing allelic frequencies in diabetic patients *vs* control and in diabetic patients *vs* centenarians.

| **TABLE S2.** Allelic association: D+Co *vs* CTR and D+Co *vs* 100+ | | | | | | | | | | | |
| --- | --- | --- | --- | --- | --- | --- | --- | --- | --- | --- | --- |
|  | | **D+Co *vs* CTR** | | | | | **D+Co *vs* 100+** | | | | |
| Gene | SNPs ID | Allele | Frequency | | p-value | OR (95% CI) | Allele | Frequency | | P_value | OR (95% CI) |
|  |  |  | D+Co CTR  N=241 N=558 | |  |  |  | D+Co  N=241 | 100+  N=229 |  |  |
| *DDAH1* | rs7521189  rs13373844 | G  C | 0.4846  0.3119 | 0.4693  0.3193 | 0.5819  0.7786 | 1.063 (0.8545-1.323)  0.9667 (0.7633-1.224) | G  C | 0.4532  0.3373 | 0.4933  0.2978 | 0.1502  0.132 | 0.8514 (0.6837-1.06)  1.2 (0.9464-1.522) |
| *IRS1* | rs2943641 | T | 0.37 | 0.3764 | 0.8148 | 0.9733 (0.7761-1.221) | T | 0.3989 | 0.3683 | 0.2623 | 1.138 (0.9076-1.428) |
| *TERC* | rs12696304 | G | 0.2891 | 0.2795 | 0.6997 | 1.049 (0.8242-1.334) | G | 0.2746 | 0.2227 | 0.03561 | 1.321 (1.018-1.714) |
| *IGF2BP* | rs4402960  rs1470579 | T  C | 0.3612  0.3423 | 0.3214  0.3036 | 0.1304  0.1405 | 1.194 (0.9488-1.502)  1.194 (0.9431-1.512) | T  C | 0.3527  0.3621 | 0.2986  0.3054 | 0.04279  0.03443 | 1.279(1.008-1.624)  1.291 (1.019-1.635) |
| *ADIPOQ* | rs266729  rs1063539 | G  C | 0.2478  0.08543 | 0.2351  0.1128 | 0.59  0.1301 | 1.072 (0.8323-1.381)  0.7344 (0.4917-1.097) | G  C | 0.2455  0.1082 | 0.3164  0.1527 | 0.005224  0.01602 | 0.703 (0.5486-0.9008)  0.6734 (0.4874-0.9304) |
| *hTERT* | rs2736098  rs2735940  rs2736109  rs401681 | A  C  A  T | 0.3191  0.4009  0.4085  0.4279 | 0.3058  0.4147  0.43  0.4129 | 0.6267  0.6145  0.4377  0.5827 | 1.064 (0.8293-1.364)  0.9444 (0.7558-1.18)  0.9153 (0.7321-1.144)  1.064 (0.8532-1.326) | A  C  A  T | 0.4088  0.4203  0.4099  0.2952 | 0.4051  0.4276  0.4182  0.2977 | 0.8929  0.7912  0.7666  0.9224 | 1.016 (0.8099-1.274)  0.9703 (0.7765-1.213)  0.9665 (0.7721-1.21)  0.9879 (0.7728-1.263) |
| *EPO* | rs1617640  rs507392  rs551238 | G  C  C | 0.3079  0.319  0.3048 | 0.3424  0.3513  0.3418 | 0.1872  0.2281  0.1581 | 0.8543 (0.676-1.08)  0.865 (0.6832-1.095)  0.8443 (0.6674-1.068) | G  C  C | 0.3312  0.3229  0.3246 | 0.3226  0.2707  0.32 | 0.7478  0.05052  0.8606 | 1.04 (0.8197-1.319)  1.285 (0.9991-1.652)  1.021 (0.8076-1.291) |
| *TCF7L2* | **rs7903146** | **T** | **0.4612** | **0.3738** | **1.66*10^-3^** | **1.434 (1.145-1.795)** | **T** | **0.45** | **0.3145** | **1.07*10^-6^** | **1.784 (1.412-2.253)** |
| *CAT* | rs1001179 | A | 0.1812 | 0.211 | 0.1918 | 0.8276 (0.6228-1.1) | A | 0.05495 | 0.04167 | 0.2878 | 1.337 (0.781-2.29) |
| *KCNJ11* | rs5215 | C | 0.3194 | 0.3367 | 0.5104 | 0.9245 (0.7318-1.168) | C | 0.3565 | 0.3496 | 0.7949 | 1.031 (0.8198-1.296) |
| *KCNQ1* | rs2237892 | T | 0.05507 | 0.03895 | 0.1571 | 1.438 (0.8673-2.384) | T | 0.2158 | 0.1895 | 0.2554 | 1.177 (0.8886-1.559) |
| *HIF-1α* | rs11549465 | T | 0.1507 | 0.1495 | 0.9518 | 1.009 (0.7442-1.369) | T | 0.1452 | 0.125 | 0.298 | 1.189 (0.8582-1.646) |
| *FTO* | rs8050136  rs9939609 | A  A | 0.4846  0.48 | 0.4402  0.433 | 0.1088  0.09076 | 1.196 (0.9609-1.488)  1.209 (1.209-1.506) | A  A | 0.4731  0.4683 | 0.4951  0.4409 | 0.4474  0.3297 | 0.9157 (0.7295-1.149)  1.117 (0.8943-1.395) |

**Table S2**. Allelic association comparing allelic frequencies in diabetic patients with complications *vs* control and diabetic patients with complications *vs* centenarians.

**TABLE S3.** Genotypic association: D *vs* CTR and D+Co *vs* CTR

|  | | **D *vs* CTR** | | | | | | | **D+Co *vs* CTR** | | | | | |
| --- | --- | --- | --- | --- | --- | --- | --- | --- | --- | --- | --- | --- | --- | --- |
| **Gene** | **SNPs ID** | Genotype | Model | Frequency  D Ctrl | | | p-value | OR (95% CI) | Genotype | Model | Frequency  D+C Ctrl | | p-value | OR (95% CI) |
| ***DDAH1*** | **rs7521189**  **rs13373844** | G/A  C/C | Overdominant  Recessive | 55.4  8.6 | 48.6  12.3 | | 0.02250  0.04305 | 0.76 (0.60-0.96)  1.49 (1.01-2.21) | G/G  C/A | Recessive  Overdominant | 19.3  46.8 | 21.8  41.2 | 0.4192  0.1547 | 1.17 (0.80-1.71)  0.80 (0.58-1.09) |
| ***IRS1*** | **rs2943641** | T/T | Recessive | 12.9 | 16.2 | | 0.1188 | 1.31 (0.93-1.83) | C/T | Overdominant | 49.5 | 39.9 | 0.01494 | 0.68 (0.50-0.93) |
| ***TERC*** | **rs12696304** | C/G | Overdominant | 39.4 | 37.4 | | 0.5009 | 0.92 (0.72-1.17) | C/G | Overdominant | 39.4 | 42.2 | 0.4629 | 1.12 (0.82-1.53) |
| ***IGF2BP*** | **rs1470579**  **rs4402960** | C/A-C/C | Dominant  Log-additive | 49.2 | 56.5 | | 0.01668  0.04176 | 1.34 (1.05-1.71)  1.20 (1.01-1.43) | G/T-T/T  C/A-C/C | Dominant  Dominant | 53.0  49.3 | 60.7  56.5 | 0.04895  0.07226 | 1.37 (1.00-1.87)  1.33 (0.97-1.83) |
| ***ADIPOQ*** | **rs266729**  **rs1063539** | G/C-G/G  C/C | Dominant  Recessive | 41.2  1.7 | 42.8  1.4 | | 0.5782  0.6249 | 1.07 (0.84-1.36)  0.78 (0.29-2.11) | G/C-G/G  C/G-C/C | Dominant  Dominant | 41.1  20.7 | 43.1  15.4 | 0.6001  0.09905 | 1.09 (0.80-1.48)  0.70 (0.45-1.08) |
| ***h TERT*** | **rs2736098**  **rs2735940**  **rs2736109**  **rs401681** | A/A  C/C  A/A  T/C | Recessive  Recessive  Recessive  Overdominant | 9.5  16.6  17.6  47.1 | 7.4  15.0  14.9  51.1 | | 0.2406  0.4584  0.2279  0.1862 | 0.77 (0.49-1.20)  0.88 (0.64-1.22)  0.82 (0.59-1.13)  1.17 (0.93-1.48) | A/G-A/A  C/C  T/C | Dominant  Recessive  Log-additive  Overdominant | 51.6  16.5  47.2 | 53.8  14.0  53.2 | 0.6100  0.3812  0.3789  0.1224 | 1.09 (0.78-1.5)  0.82 (0.53-1.28)  0.90 (0.72-1.14)  1.27 (0.94-1.73) |
| ***EPO*** | **rs1617640**  **rs507392**  **rs551238** | G/T  C/T  C/A | Overdominant  Overdominant  Overdominant | 46.8  48.8  47.0 | 42.4  44.7  42.9 | | 0.1352  0.1784  0.1644 | 0.84 (0.66-1.06)  0.85 (0.67-1.08)  0.85 (0.67-1.07) | G/T-G/G  C/T-C/C  C/A-C/C | Dominant  Dominant  Dominant | 57.8  59.6  57.8 | 50.9  54.1  51.1 | 0.07615  0.1574  0.08666 | 0.76 (0.56-1.03)  0.80 (0.58-1.09)  0.76 (0.56-1.04) |
| ***TCF7L2*** | **rs7903146** | **T/C-T/T** | **Dominant** | **58.9** | **70** | **1.415*10^-4^** | | **1.63 (1.26-2.09)** | **T/C-T/T** | **Dominant** | **58.8** | **71.9** | **5.96*10^-4^** | **1.79 (1.28-2.52)** |
| ***CAT*** | **rs1001179** | A/A | Recessive | 3.7 | 4.2 | | 0.6511 | 1.15 (0.62-2.14) |  | Log-additive |  |  | 0.2111 | 0.83 (0.62-1.11) |
| ***KCNJ11*** | **rs5215** | C/C | Recessive | 10.9 | 14.6 | | 0.06259 | 1.40 (0.98-2.00) | T/C | Overdominant | 45.3 | 38.2 | 0.06727 | 0.75 (0.54-1.02) |
| ***KCNQ1*** | **rs2237892** | T/C | Codominant | 7.7 | 9.9 | | 0.08419 | 1.32 (0.87-2) | T/C | Codominant | 7.8 | 10.9 | 0.162 | 1.46 (0.87-2.45) |
| ***HIF-1α*** | **rs11549465** | T/C | Overdominant | 25.0 | 24.0 | | 0.6894 | 0.95 (0.72-1.24) | T/C | Overdominant | 24.9 | 26.4 | 0.6612 | 1.08 (0.76-1.54) |
| ***FTO*** | **rs8050136**  **rs9939609** | A/A  A/A | Recessive  Recessive | 18.4  17.8 | 23.2  23.0 | | 0.04919  0.03271 | 1.34 (1.00-1.79)  1.38 (1.03-1.85) | A/A  A/A | Recessive  Recessive | 18.4  17.9 | 24.3  24.7 | 0.06266  0.03317 | 1.43 (0.99-2.06)  1.51 (1.04-2.18) |

**Table S3**. Genotypic associations analysis comparing diabetic patients (N = 562) and control (N = 558) and diabetic individuals with complications (N = 241) and controls (N = 558).

**TABLE S4. Genotypic association: D *vs* 100+ and D+Co *vs* 100+**

|  | | **D *vs* 100+** | | | | | | **D+Co *vs* 100+** | | | | | |
| --- | --- | --- | --- | --- | --- | --- | --- | --- | --- | --- | --- | --- | --- |
| **Gene** | **SNPs ID** | Genotype | Model | Frequency  D 100+ | | p_value | OR (95% CI) | Genotype | Model | Frequency  D+Co 100+ | | p-value | OR |
| ***DDAH1*** | **rs7521189**  **rs13373844** | G/G | Recessive  Log-additive | 21.0 | 27.1 | 0.0704  0.1393 | 0.72 (0.50-1.02)  1.19 (0.94-1.50) | G/A  C/A-C/C | Overdominant  Dominant | 53.3  52.2 | 44.4  49.3 | 0.05967  0.5427 | 1.43 (0.98-2.06)  1.12 (0.78-1.62) |
| ***IRS1*** | **rs2943641** | T/T | Recessive | 16.2 | 12.1 | 0.1314 | 1.42 (0.89-2.24) | C/T | Overdominat | 39.9 | 49.6 | 0.03912 | 0.68 (0.47-0.98) |
| ***TERC*** | **rs12696304** |  | Log-additive |  |  | 0.03979 | 1.30 (1.01-1.67) | C/G-G/G | Dominant | 50.0 | 38.2 | 0.01133 | 1.62 (1.11-2.35) |
| ***IGF2BP*** | **rs1470579**  **rs4402960** | C/A-C/C  G/T – T/T | Dominant  Dominant | 56.5  58.8 | 47.5  49.3 | 0.02337  0.01666 | 1.44 (1.05-1.97)  1.47 (1.07-2.01) | C/A  G/T | Overdominat  Overdominat | 44.8  49.3 | 35.3  37.6 | 0.03990  0.01157 | 1.49 (1.02-2.18)  1.62 (1.11-2.36) |
| ***ADIPOQ*** | **rs266729**  **rs1063539** | G/C  C/G | Overdominant  Overdominant | 36.6  18.9 | 50.7  27.9 | 4.196*10^-4^  0.007277 | 0.56 (0.41-0.77)  0.60 (0.42-0.87) | C/G - G/G  C/G | Dominat  Overdominant | 43.1  13.9 | 57.0  27.9 | 0.003584  0.000376 | 0.57 (0.39-0.83)  0.42 (0.26-0.69) |
| ***h TERT*** | **rs2736098**  **rs2735940**  **rs2736109**  **rs401681** | A/G  C/T  A/A | Overdominant  Overdominant  Recessive  Log-additive | 44.2  51.8  14.9 | 39.5  43.1  17.3 | 0.2466  0.02892  0.4147  0.7862 | 1.21 (0.88-1.67)  1.42 (1.04-1.95)  0.84 (0.55-1.28)  0.97 (0.77-1.22) | A/G  C/T  A/A  T/C | Overdominat  Overdominant  Recessive  Overdominant | 43.7  52.0  13.8  53.2 | 39.5  43.1  17.3  51.1 | 0.3867  0.05981  0.3082  0.6526 | 1.19 (0.80-1.75)  1.43 (0.98-2.08)  0.77 (0.46-1.28)  1.09 (0.75-1.57) |
| ***EPO*** | **rs1617640**  **rs507392**  **rs551238** | C/C  C/C | Log-additive  Recessive  Recessive | 10.8  11.0 | 9.2  10.2 | 0.0507  0.5237  0.7406 | 1.28 (1.00-1.64)  1.19 (0.70-2.03)  1.09 (0.66-1.81) | G/G  C/T  C/A-C/C | Recessive  Overdominant  Dominant | 9.9  44.6  51.1 | 6.8  53.8 | 0.2449  0.7541  0.2168 | 1.50 (0.75-3)  0.94 (0.65-1.37)  0.90 (0.62-1.30) |
| ***TCF7L2*** | **rs7903146** |  | **Log-additive** |  |  | **9.066*10^-7^** | **1.78 (1.41-2.26)** |  | **Log-additive** |  |  | **5.473*10^-6^** | **1.89 (1.43-2.50)** |
| ***CAT*** | **rs1001179** |  | Log-additive |  |  | 0.2445 | 1.18 (0.89-1.58) | G/A | Overdominant | 31.1 | 32.4 | 0.7581 | 0.94 (0.63-1.40) |
| ***KCNJ11*** | **rs5215** | C/C | Recessive | 14.6 | 12.4 | 0.4101 | 1.21 (0.76-1.92) | C/T | Overdominant | 38.2 | 45.1 | 0.1315 | 0.75 (0.52-1.09) |
| ***KCNQ1*** | **rs2237892** | T/C | Overdominant | 9.9 | 6.5 | 0.1229 | 1.59 (0.86-2.92) |  | Log-additive |  |  | 0.06171 | 1.31 (0.71-2.41) |
| ***HIF-1α*** | **rs11549465** | T/C | Overdominant | 24.0 | 19.6 | 0.1828 | 1.29 (0.88-1.90) | T/C | Overdominant | 26.4 | 19.6 | 0.08627 | 1.47 (0.94-2.28) |
| ***FTO*** | **rs8050136**  **rs9939609** | C/A-A/A  A/T-A/A | Dominant  Dominant | 71.5  70.7 | 74.4  66.8 | 0.4224  0.2983 | 0.86 (0.60-1.24)  1.20 (0.86-1.67) | C/A-A/A | Dominant  Log-additive | 72.6 | 74.4 | 0.6762  0.2553 | 0.91 (0.59-1.40)  1.16 (0.90-1.50) |

**Table S4**. Genotypic associations analysis comparing diabetic individuals (N = 562) and centenarians (N = 229) and diabetic individuals with complications (N = 241) and centenarians (N = 229).

**TABLE S5**. List of genes and SNPs considered in the present study.

| **Genes** | **SNPs (rs)** |
| --- | --- |
| ***DDAH1*** | rs7521189, rs669173, rs13373844 |
| ***IRS1*** | rs2943641 |
| ***TERC*** | rs12696304 |
| ***IGF2BP*** | rs4402960, rs1470579 |
| ***ADIPOQ*** | rs266729, rs1063539 |
| ***h TERT*** | rs2736098, rs2853669, rs2735940, rs2736109, rs401681 |
| ***VEGF*** | rs3025021, rs10434 |
| ***SOD2*** | rs4880 |
| ***EPO*** | rs1617640, rs507392, rs551238 |
| ***SLC30A8*** | rs13266634, rs16889462 |
| ***TCF7L2*** | rs7901695, rs7903146 |
| ***CAT*** | rs1001179 |
| ***KCNJ11*** | rs5215 |
| ***KCNQ1*** | rs2237892 |
| ***HIF-1alpha*** | rs11549465 |
| ***FTO*** | rs8047395, rs8050136, rs9939609 |

**Table S5**. Underlined rs were excluded from the analysis after quality control.
